# In-Situ pH-Sensitive Fibers via the Anchoring of Bromothymol Blue on Cellulose Grafted with Hydroxypropyltriethylamine Groups via Adsorption

**DOI:** 10.3390/polym10070709

**Published:** 2018-06-27

**Authors:** Lele Cao, Tieqiang Liang, Xipeng Zhang, Wenbo Liu, Jian Li, Xianxu Zhan, Lijuan Wang

**Affiliations:** 1 Key Laboratory of Bio-Based Materials Science and Technology of Ministry of Education, Northeast Forestry University, Harbin 150040, China; 18846170635@163.com (L.C.); ltq@nefu.edu.cn (T.L.); hljwbo@nefu.edu.cn (W.L.); nefulijian@163.com (J.L.); 2 Research Center of Wood Bionic Intelligent Science, Northeast Forestry University, Harbin 150040, China; 3 School of Environment, Harbin institute of Technology, Harbin 150040, China; 96315zh@163.com; 4 Dehua TB New Decoration Material CO., LTD, Deqing 313200, China; zhanxianxu@126.com

**Keywords:** cellulose fiber, cationic modification, anchoring, bromothymol blue, in-situ pH-sensitive

## Abstract

In-situ pH-sensitive cellulose fibers (IS-pH-SCF) were prepared by anchoring bromothymol blue (BTB) onto cellulose fibers (CF) modified with hydroxypropyltriethylamine (HPTTL) groups. Fourier transform infrared and X-ray photoelectron spectrum analyses demonstrated that the HPTTL groups were grafted onto the CF. X-ray diffraction proved that cellulose I in the CF transformed into cellulose II after quaternization. Scanning electron microscopy suggested that the quaternized CF (QCF) surface was clean and uniformly ridged. The adsorption of BTB onto QCF was carried out via batch adsorption experiments. A kinetic study illustrated that the adsorption was a spontaneous process and described well by pseudo-second-order, Freundlich and Temkin isotherms. The activation energy for the BTB adsorption onto QCF was 52.89 kJ/mol, which proved that the BTB adsorption onto QCFs was chemically controlled. The pH response demonstrated that the IS-pH-SCF was highly sensitive to pH, with an obvious color change for pH 4 to 8. The release tests showed that BTB was anchored on QCFs and that no BTB was released. IS-pH-SCF has a potential use for indicating pH changes in food.

## 1. Introduction

Foods are rich in nutrients and are the most basic materials for human survival. Food safety is directly related to human physical health and life safety, and is critical to ensure both economic and social stability. Food quality deteriorates because of moisture, light, microbial reproduction during transportation, and storage. Food spoilage could lead to a reduction or loss of nutritional value and even cause food poisoning, with a potential for great harm and significant economic losses. Improvements in living standards have resulted in people paying increased attention to food safety. The traditional food quality test is based on a chemical analysis, which requires the destruction of foods, expensive analytical instruments, professional operators, and extended times [1]. Such analyses are not always feasible because the detection is not readily accessible by the public. To overcome these disadvantages, intelligent packaging materials and indicators have been favored by scholars for the real-time detection of food quality [2]. In general, the spoilage of foods may be accompanied by pH changes. For example, protein-rich foods (fish and pork, for example) release organic amines during the spoilage, which results in an increase in pH [3]. Polysaccharide-rich foods release carbon dioxide and other acidic gases, which leads to a reduction in pH. In recent years, pH-sensitive sensors [4,5,6,7,8,9,10] and pH-sensitive packaging films [11,12,13,14] have been reported. Synthetic dyes are highly sensitive and have obvious color changes for tiny pH changes, and this phenomenon has been used by many researchers. A pH dye-based indicator, prepared by coating a mixed solution of bromothymol blue and methyl red on a film that was prepared with nylon with linear low-density polyethylene, was applied to monitor a golden drop (a premium dessert produced in Thailand), and proved that it can serve as a real-time indicator for spoilage via color changes [15]. Lee [3] prepared a gas-freshness indicator composed of bromothymol blue-phenol red, which was used to indicate the freshness of fish by obvious color changes based on the quantity of volatile amines. Although synthetic dyes are much more sensitive to pH changes, their inedibility makes them potentially harmful for human health when they migrate from the matrices to food. Therefore, researchers have focused on natural pigments extracted from fruits or vegetables [16,17,18] to prepare pH indicators or pH sensitive packaging materials [19,20,21]. Chen reported that a pH sensitive fabric dyed by using turmeric showed a significant color change when the pH changed from neutral to alkaline [22]; however, no obvious color change resulted when the pH changed from neutral to acidic. Ma [19] prepared an intelligent packaging film by incorporating anthocyanins that were extracted from mulberry to monitor the putrefaction of fish. The color changed to green when the fish was putrid. However, the color change was not sensitive or not sufficiently accurate to indicate the inflection point from freshness to slight spoilage of the fish. As a result of their chemical structures, natural pigments are not accurately sensitive to slight pH changes. To obtain highly pH-sensitive fibers, pH sensitive synthetic dyes are required and migration must be prevented via firm interactions. To our knowledge, no report exists on in-situ pH-sensitive cellulose fibers (CFs) via the anchoring of pH-sensitive synthetic dyes.

**Figure 1:**
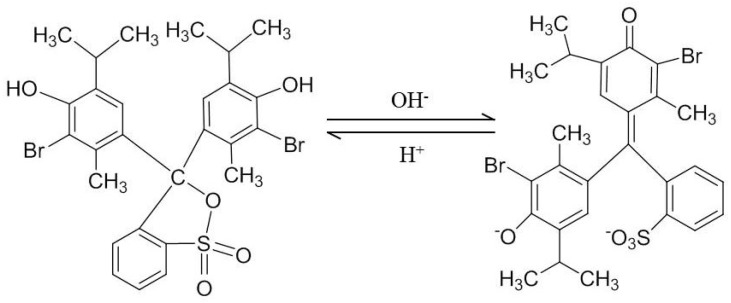
Structural changes of bromothymol blue in acid and alkaline solutions.

Bromothymol blue (BTB) is a pH-sensitive synthetic dye that is used extensively in indicators [23,24,25]. As shown in Figure 1, it is negatively charged in alkaline solution. Cellulose fiber (CF) is a rich, renewable, biodegradable and non-toxic material composed of a linear chain of β-(1→4)-linked d-glucopyranosyl units [26]. CF is insoluble in water and any other common organic solvents [27]. It is a favorite carrier to anchor pH-sensitive synthetic dyes. However, OH-rich CF cannot anchor BTB because the interactions between the molecules are too weak. Therefore, a cationic modification is necessary for CF to anchor BTB in alkaline conditions through electrostatic attraction.

In this study, CFs were positively charged via a cationic modification in which quaternary ammonium groups were grafted onto cellulose molecules. In-situ pH-sensitive CFs (IS-pH-SCF) were prepared by anchoring BTB onto quaternized CFs (QCF) in a NaOH solution. The QCF and IS-pH-SCF were analyzed by Fourier transform infrared (FTIR) spectroscopy, X-ray photoelectron spectroscopy (XPS), X-ray diffraction (XRD), and scanning electron microscopy (SEM). QCF was tested as an adsorbent for BTB from a NaOH solution. The effects of the QCF dosage and contact time were investigated. The kinetics and isotherms of the adsorption process were studied. The pH-sensitivity of the IS-pH-SCF and the non-release of BTB were measured.

## 2. Materials and Methods 

### 2.1. Materials

Bleached softwood kraft pulp was used as a cellulose fiber (CF) and offered by Henfeng Paper Co., Ltd. (Mudanjiang, China). Bromothymol blue (BTB) was supplied by the Guangfu Chemical Research Institute (Tianjin, China). All other chemicals, including sodium hydroxide, epoxy chloropropane, triethylamine, hydrochloric acid, absolute ethyl alcohol and buffer solutions (pH 4 to 8), were supplied by Yongda Chemical Reagent Co., Ltd. (Tianjin, China). All chemicals were of analytical grade and used without purification. 

### 2.2. Preparation and Characterization of Quaternized Cellulose Fiber (QCF)

#### 2.2.1. Preparation of the QCF

QCF was prepared according to the literature with slight modifications [28]. The CF was ground to a powder with a size of 125~180 µm. The CF powder (5 g) and 125 mL of a NaOH solution (20% *w*/*w*) were stirred at 500 rpm and 25 °C for 2 h. A NaOH solution (125 mL, 10% *w/w*) and 60 mL of epoxy chloropropane were added into the flask after the solution had been removed by filtration. The mixture was heated to 65 °C and stirred at 500 rpm for 7 h. 70 mL of triethylamine/absolute ethyl alcohol solution (34% *v/v*) was added into the flask after a solid-liquid separation, and the mixture was stirred at 500 rpm and 75 °C for 4 h. Absolute ethanol was used to wash the resulting product to remove the unreacted triethylamine. Then, the product was washed successively with 0.1 M NaOH, 0.1 M HCl and distilled water until it reached a pH of 7. The QCF was dried at 65 °C for 10 h.

#### 2.2.2. Characterization of the QCF

The FTIR spectra of the CF and the QCF were measured by using a Nicolet 6700 spectrometer (ThermoFischer, Waltham, MA, USA), and tests were conducted from 500 to 4000 cm^−1^ with a resolution of 4 cm^−1^. An XPS analyzer with K-Alpha (Thermo, VGS, Waltham, MA, USA) was employed to detect the X-ray photoelectron spectroscopy. The XRD patterns of the CF and the QCF were acquired by using a D/MAX-2500 diffractometer (Cu-K α target, 40 kV, 30 mA) at 1200 W (Rigaku, Tokyo, Japan). A Hitachi SU-70 microscope (Hitachi, Ibaraki, Japan) was employed to analyze the surface morphology of the CF and the QCF. A thin layer of gold was deposited on the surface of the samples before the observation.

### 2.3. Adsorption Experiments

The adsorption of BTB on the QCF in the dilute alkaline solution (0.1 M NaOH) and the effects of both the QCF dosage and the adsorption time on the adsorption were investigated by batch adsorption experiments. The standard curve of BTB in a 0.1 M NaOH solution was obtained by using an ultraviolet–visible spectrophotometer (UV-2600, Shimadzu, Kyoto, Japan). For each adsorption experiment, 50 mL of BTB/NaOH solution with 1000 mg/L and a certain amount of QCF were added into a beaker under magnetic stirring of 200 rpm for different times until the adsorption reached an equilibrium. The mixture was separated by using a benchtop high-speed centrifuge (TG16-WS, Changsha, China) at 10,000 r/min for 6 min. Subsequently, the residual BTB/NaOH solution was diluted 50 times with a 0.1 M NaOH solution, and then the absorbance was measured with the ultraviolet–visible spectrophotometer at 615 nm. The concentration of BTB was calculated according to the standard curve. The adsorption capacity at time t (q_t_, mg/g) and the equilibrium (q_e_, mg/g) are obtained from the following equations:

q_e_ = [(C_0_ − C_e_)/m] × V (1)

q_t_ = [(C_0_ − C_t_)/m] × V (2)

where C_0_, C_e_ and C_t_ (mg/L) denote the concentration of the BTB/NaOH solution at initial, equilibrium and t (min), respectively. V (L) represents the volume of BTB/NaOH solution and m (g) presents the QCF dosage.

### 2.4. Adsorption Kinetics and Isotherms

#### 2.4.1. Kinetic Models

The adsorption experiments were performed by magnetically stirring 100 mg of QCF and 50 mL of BTB/NaOH solution with 1000 mg/L at 200 rpm for different time intervals at various temperatures to explore the kinetics. The adsorption process was analyzed via pseudo-first-order and pseudo-second-order models [29]. In general, the pseudo-first-order is used to elaborate the initial stage of the adsorption process and can be shown as:

ln(q_e_ − q_t_) = lnq_e1_ − K_1_t (3)

where q_e_ and q_t_ (mg/g) indicate the quality of BTB adsorbed onto the QCF at equilibrium and t (min), respectively. The q_e1_ (mg/g) denotes the theoretical value of BTB adsorbed onto the QCF at equilibrium, and K_1_ (1/min) represents the rate constant.

The pseudo-second-order model describes the whole process of the adsorption and conforms to the mechanism of chemical adsorption [30]. The model can be matched by the following equation:

t/q_t_ = 1/(K_2_q_e2_^2^) + t/q_e2_ (4)

where q_e2_ (mg/g) denotes the theoretical quality of BTB adsorbed onto the adsorbent at equilibrium, and q_t_ (mg/g) is denotes the theoretical quality of BTB adsorbed onto the adsorbent at time t (min). The K_2_ (g/mg min) represents the rate constant.

The activation energy Ea [31] of the adsorption can be calculated by using the following equation:

lnK_2_ = lnA − Ea/(RT) (5)

where A represents the Arrhenius factor.

#### 2.4.2. Isotherm Models

50 mL of a BTB/NaOH solution with 1000 mg/L and a certain quality (100, 200, 300, 400, 500, or 600 mg) of QCF were stirred in a magnetic stirring water bath at 200 rpm for 4 h at different temperatures (303.15, 313.15, or 323.15 K) to study the adsorption isotherm. Langmuir [32], Freundlich [33] and Temkin [34] isotherms were applied to analyze the experiment data and to help understand the adsorption process. The adsorption isotherm models can be represented as:

C_e_/q_e_ = 1/(K_L_q_m_) + C_e_/q_m_ (6)

q_e_ = K_f_ × C_e_^1/n^ (7)

q_e_ = A + B × lnC_e_ (8)

where q_m_ (mg/g) is the maximum adsorption capacity, q_e_ (mg/g) is the equilibrium adsorption capacity, C_e_ (mg/L) represents the concentration of the BTB/NaOH solution at equilibrium, and K_L_ (L/mg) denotes the Langmuir adsorption constant. K_f_ denotes the Freundlich constant and n denotes the heterogeneity factor. A and B are the Temkin dasorption isothermal constants [35].

### 2.5. Preparation and Characterization of In-Situ pH-Sensitive Cellulose Fibers (IS-pH-SCF)

#### 2.5.1. Preparation of IS-pH-SCF

The QCF (2 g) and 200 mL of a BTB/NaOH solution (1000 mg/L) were added into a beaker and stirred in a magnetic stirring water bath at 200 rpm for 4 h at 50 °C. The mixture was separated via filtration with a 200 mesh filter cloth. The isolated solids were soaked in distilled water and stirred at ~500 rpm for 5 min, followed by a vacuum filtration, and this was repeated 5 times until the filtrate was colorless and neutral. 0.1 g of IS-pH-SCFs and distilled water (50 mL) were stirred at 500 rpm to disperse the IS-pH-SCFs uniformly. Following this, the suspension was added slowly into the Buchner funnel equipped with two layers of filter paper and vacuum filtration was performed at 0.08 MPa. Circular IS-pH-SCF felts were obtained and dried at 65 °C.

#### 2.5.2. Characterization of IS-pH-SCF

The FTIR analysis and SEM observation of IS-pH-SCF were conducted by using the same methods as those for QCF.

### 2.6. pH Response of IS-pH-SCF

The circular pieces of IS-pH-SCF felts were cut into rectangles (2 cm × 2 cm). Individual buffered solutions (5 mL, pH = 4, 5, 6, 7, and 8) were placed in glass culture dishes (marked 4 to 8). The IS-pH-SCF felt was soaked in a buffer solution for 30 s, then removed with tweezers and placed on several layers of filter paper. A portable colorimeter (Xrite2600d, X-rite, Grand Rapids, MI, USA) was employed to analyze the color of the IS-pH-SCF rectangles, including L (luminosity), a (carmine-green) and b (yellow-blue). Five detections were performed for each IS-pH-SCF. The total chromatic difference (∆E) was obtained as:

(9) $$\Delta E=\sqrt{{\left(L-L_{0}\right)}^{2}+{\left(a-a_{0}\right)}^{2}+{\left(b-b_{0}\right)}^{2}}$$

where L_0_, a_0_ and b_0_ are chromatic values of the IS-pH-SCF. L, and a and b are chromatic parameters of the IS-pH-SCF at different pH values.

### 2.7. Release of BTB from IS-pH-SCF

A piece of the IS-pH-SCF was soaked in distilled water (pH = 7) for 24 h, before the IS-pH-SCF was taken out with a pair of tweezers. Subsequently, the color of the resulting distilled water was observed after the very small fibers were separated by centrifugation. An HCl solution (0.1 M) was dropped on an IS-pH-SCF felt on a piece of filter paper, and the color change of the IS-pH-SCF was observed. After 5 min, the IS-pH-SCF was removed and the color of the original location where IS-pH-SCF had been was observed before and after drying. In addition, the experiments were also conducted by using a NaOH solution (0.1 M).

## 3. Results and Discussion

### 3.1. Analyses of the QCF

**Figure 2:**
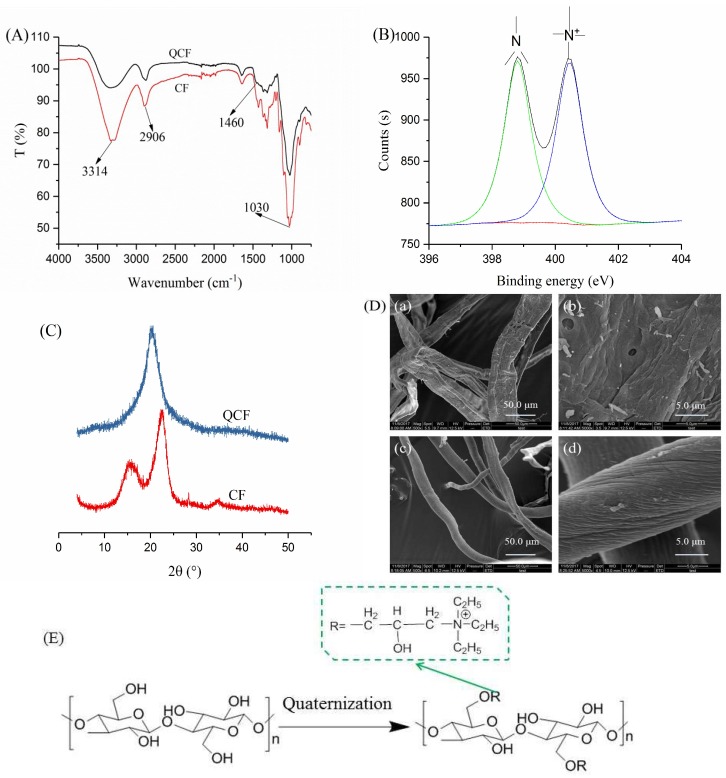
(**A**) FTIR spectra of CF and QCF; (**B**) N1s spectrum of QCF; (**C**) XRD patterns of CF and QCF; (**D**) SEM photographs of CF ((**a**) ×500, (**b**) ×5000) and QCF ((**c**) ×500, (**d**) ×5000); and (**E**) the chemical structure of QCF.

Figure 2A shows the FTIR spectra of the CF and the QCF. The band from 3695 to 2978 cm^−1^ is the O–H stretching vibration, and the band at ~2906 cm^−1^ corresponds to the C–H stretching of the –CH_2_– groups. The bands at ~1158, 1098, 1057, and 1030 cm^−1^ originated from the C–O–C stretching from the glycosidic bonds of the cellulose molecule [36]. After quaternization, the O–H stretching band weakened and moved to a high wavenumber, and a new band occurred at ~1460 cm^−1^, corresponding to the C–N stretching vibration of –N^+^(C_2_H_5_)_3_ [37]. The changes showed that the –N^+^(C_2_H_5_)_3_ has been grafted onto the cellulose skeleton. In the XPS spectrum (Figure 2B), peaks at 398.81 and 401.26 eV showed that nitrogen was present in the QCF in ternary and quaternary states [38,39], respectively. The former is likely to be an admixture of trimethylamine in the QCF preparation. The total nitrogen content of the QCF was 1.73% from the XPS analysis. The XPS results further confirmed the FTIR analysis. 

Figure 2C exhibits the XRD patterns of the CF and the QCF. The CF showed a typical cellulose I structure including peaks at 15.62°, 22.62° and 34.08°. Compared with the CF, the characteristic peaks disappeared and a new peak at 20.32° appeared in the XRD pattern of the QCF, which indicated a typical cellulose II structure, and showed that intermolecular and intramolecular hydrogen bonds of CF were destroyed during quaternization. The SEM photographs of the CF and the QCF are exhibited in Figure 2D. The CF fibers [Figure 2 (Da,Db)] were ribbon-like with impurities, showing a rough and wrinkled surface. After quaternization, the impurities disappeared and the surface was uniformly ridged [Figure 2 (Dc,Dd)]. The chemical structure of the QCF can be described as shown in Figure 2E based on the above analysis.

### 3.2. Effect of Contact Time

**Figure 3:**
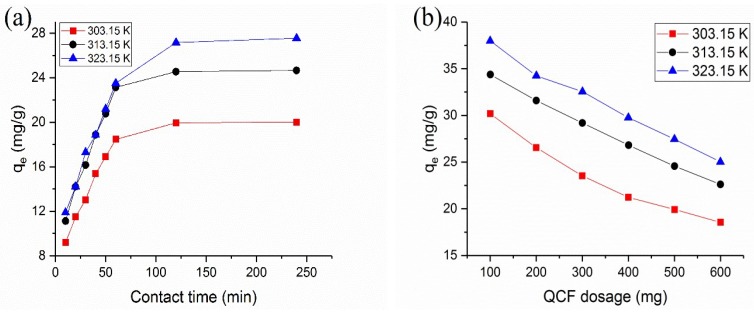
Effects of (**a**) contact time and (**b**) QCF dosage on the BTB adsorption onto QCF.

The effects of the adsorption time on the BTB adsorption onto the QCF at 303.15, 313.15 and 323.15 K are exhibited in Figure 3a. The entire adsorption process can be distributed into three sections: a rapid adsorption phase (0~60 min) which shows a liner growth trend; a slow adsorption phase (60~90 min) and an equilibrium stage from 120 to 240 min. The increase in the adsorption rate during the primary stage may have resulted from the higher force and sufficient availability of active sites for BTB to transfer to the QCF. The amount of active sites decreased rapidly with an increase in contact time, which resulted in a reduction in the adsorption rate. The equilibrium adsorption capability increased as the temperature increased, which demonstrated that a higher temperature benefited the BTB adsorption on the QCF.

### 3.3. Effect of Adsorbent Dosage

As can be seen from Figure 3b, the equilibrium adsorption capacity decreased with an increase of the QCF dosage, showing a liner reduction. The increased quality of the QCF could provide more active sites for BTB adsorption, and the adsorption reached saturation with a small amount of the QCF. The equilibrium adsorption capacity gradually increased as the temperature increased, which demonstrated that a high temperature promoted the adsorption. 

### 3.4. Adsorption Kinetics

**Figure 4:**
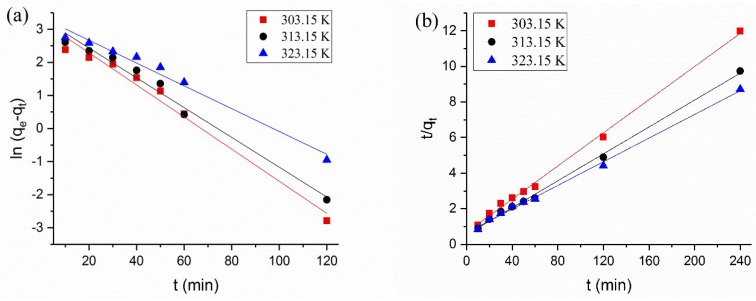
Fitting curves of (**a**) pseudo-first-order; and (**b**) pseudo-second-order.

Pseudo-first-order [40] and pseudo-second-order [41] models were applied to explore the adsorption process. The fitted curves and parameters of the two models are shown in Figure 4 and Table 1. The linearity of the pseudo-first-order model was poor, and the difference between the calculated q_e_ (q_e,cal_) and experimental q_e_ (q_e,exp_) values was large. This disparity showed that the adsorption of BTB on the QCF cannot be accurately described with the pseudo-first-order kinetic model. The R^2^ of the pseudo-second-order at three experimental temperatures were 0.998, 0.997 and 0.997, which indicated excellent linearity. The difference between the calculated q_e_ (q_e,cal_) and the experimental q_e_ (q_e,exp_) is smaller. Therefore, the pseudo-second-order can describe the adsorption process well.

The activation energy of adsorption was calculated from the Arrhenius equation. In general, the values of the activation energy can be applied to distinguish whether the adsorption is physically (4~40 kJ/mol) or chemically controlled (higher than 40 kJ/mol) [42]. The activation energy for the BTB adsorption onto the QCF was 52.89 kJ/mol, which proved that the BTB adsorption was chemically controlled. The interaction between the QCF and BTB was very strong and the strategy for anchoring BTB on cationic cellulose was successful.

### 3.5. Adsorption Isotherms

**Figure 5:**
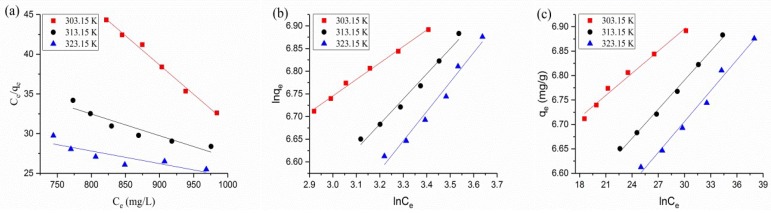
Fitting curves of (**a**) Langmuir; (**b**) Freundlich; and (**c**) Temkin isotherms.

Adsorption isotherms can tell us how the adsorption conducts and help us analyze the interaction between the adsorbate and the adsorbent [43,44]. Langmuir, Freundlich and Temkin isotherms were tested to investigate the best description for the BTB sorption equilibrium, and to help understand the adsorption mechanism. The fitting curves and parameters of each isotherm are listed in Figure 5 and Table 2. The parameters of the Langmuir isotherm showed that the values of q_m_ and K_L_ are negative, which indicated that the adsorption of BTB onto the QCF did not fit the Langmuir model well. However, the values of the correlation coefficient (R^2^) were higher than 0.96 for the Freundlich and Temkin isotherm models, which described the BTB adsorption onto the QCF well. 

### 3.6. Analyses of IS-pH-SCF

**Figure 6:**
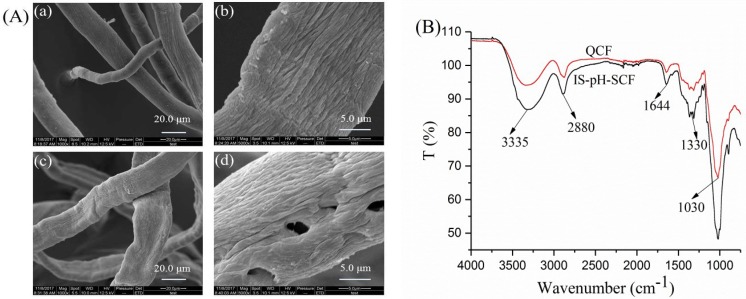
(**A**) SEM of QCF ((**a**) ×1000, (**b**) ×5000)) and IS-pH-SCF ((**c**) ×1000, (**d**) ×5000); (**B**) FTIR spectra of QCF and IS-pH-SCF.

The SEM photographs in Figure 6A show that the surface of IS-pH-SCF was similar to that of the QCF due to only ~4.0% of BTB being adsorbed on the surface, which resulted in little effect. As shown in Figure 6B, the FTIR spectra of the QCF and IS-pH-SCF were very similar. For the QCF, the band at ~3335 cm^−1^ was ascribed to the O–H. The band at ~2880 cm^−1^ was attributed to the C–H for –CH_2_– groups. The bands at ~1158, 1098, 1057, and 1030 cm^−1^ corresponded to the C–O–C from the glycosidic bonds of the cellulose molecule. After adsorption, the strength of the O–H band was enhanced and moved to a low wavenumber. The band at 2880 cm^−1^ became stronger, which indicated that the new –CH_2_– groups had been introduced into the QCF structure. A new peak at ~1330 cm^−1^ indicated the S=O stretching, and showed that the BTB had been included in the IS-pH-SCF structure. From the adsorption experiments, the maximum adsorption capacity was 38 mg/g for 1000 mg/L of BTB/NaOH solution and a dosage of 100 mg for 4 h. The BTB content of IS-pH-SCF is 0.038% (*w/w*).

### 3.7. pH Response of IS-pH-SCF

The response of the IS-pH-SCF at different pH values was shown in Table 3. The IS-pH-SCF color changed obviously when exposed to buffer solutions with a pH range from 4 to 8. The IS-pH-SCF was orange at pH 4, reddish-brown at pH 5, yellow-green at pH 6, purplish-blue at pH 7, and dark-blue at pH 8. The buffer solution also seems to exhibit a corresponding color; the reason is that the IS-pH-SCF felt was made of short fibers (~150 µm) whose intermolecular interactions are particularly weak and which will disperse once immersed in a buffer solution. The color parameters of the IS-pH-SCF were also tested for responses in different pH buffer solutions, as shown in Table 3. The b value of the IS-pH-SCF decreased rapidly from 72.510 to −0.152, which suggested that the color changed from yellow to blue. The values of a decreased significantly with an increase in pH, which indicated that the IS-pH-SCF changed from red to green. The L values exhibited a decreasing tend, which showed that the lightness of IS-pH-SCF decreased gradually with an increase in pH. These results agreed with the photographs of the IS-pH-SCF in Table 3. In general, color changes could be detected with the naked eye when the total color change (∆E) exceeded 5 [45]. The ∆E at each pH exceeded 5, which showed that the IS-pH-SCF was highly sensitive to pH values from 4 to 8.

### 3.8. Release of BTB from IS-pH-SCF

**Figure 7:**
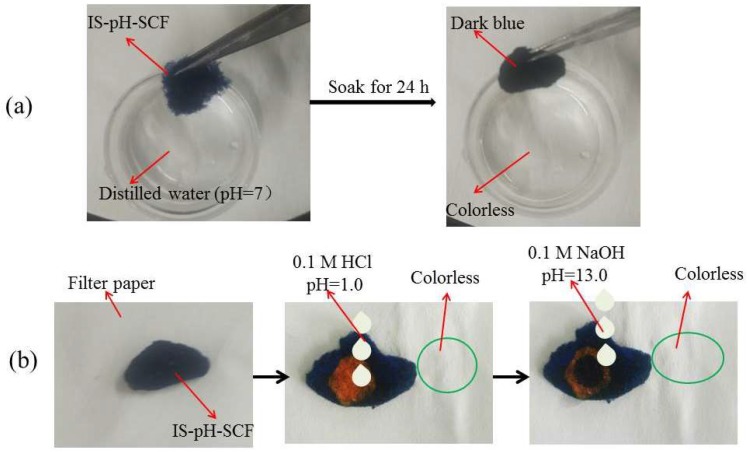
Release tests (**a**) in distilled water and (**b**) dropping acid or alkaline solutions.

The release of BTB from IS-pH-SCF in distilled water and dropping acid or alkaline solutions was investigated. As shown in Figure 7a, the color of the IS-pH-SCF did not change and the resulting distilled water after centrifugation was still colorless after the IS-pH-SCF was soaked for 24 h, which demonstrated that no BTB escaped from the fibers into distilled water. The IS-pH-SCF turned orange after making contact with the HCl solution. Subsequently, the color returned to the initial dark-blue immediately after a NaOH solution was dropped on the color-changed part. The filter paper under the IS-pH-SCF was colorless, as shown in Figure 7b, which further indicated that no BTB was released from the IS-pH-SCF. The results show that BTB successfully anchored on cationic cellulose fibers and that the fibers loading BTB are in-situ sensitive to pH change.

## 4. Conclusions

IS-pH-SCF was prepared by anchoring BTB molecules on a cationic cellulose fiber through adsorption. It was proven by FTIR and XPS analyses that the cationic modification by grafting hydroxypropyltriethylamine groups onto CFs was completed. SEM observations showed that the QCF surface became uniformly wrinkled. QCF was used as an adsorbent to anchor BTB in the alkaline solution. A higher temperature favored BTB adsorption onto the QCF. The maximum adsorption capacity was 38 mg/g for 1000 mg/L of BTB/NaOH solution and a dosage of 100 mg for 4 h. The adsorption of BTB on the QCF followed pseudo-second-order kinetics, and Freundlich and Temkin isotherms. The IS-pH-SCF exhibited a different color for each pH value from 4 to 8. Simultaneously, the BTB was not released during the pH response, which conformed that the BTB was anchored firmly on the cationic cellulose fiber. This study provides a new path for fabricating a pH-sensitive fiber that could be used to monitor the freshness of food without any pollution.

## Figures and Tables

**Table 1:** Kinetic parameters of the adsorption BTB onto QCF.

Parameters	Temperature (K)		
	303.15	313.15	323.15
q_e,exp_ (mg/g)	21.016	24.661	27.549
Pseudo-first-order			
K_1_ (min^−1^)	0.034	0.045	0.049
q_e,cal_ (mg/g)	28.703	28.022	25.972
R^2^	0.975	0.979	0.975
Pseudo-second-order			
K_2_ (mg·g^−1^·min^−1^)	0.0032	0.0026	0.0017
q_e,cal_ (mg/g)	21.459	26.316	28.303
R^2^	0.998	0.997	0.997
Ea (kJ·mol^−1^)		52.89	

**Table 2:** Isotherm parameters of the adsorption BTB onto QCF.

Parameters	Temperature (K)		
	303.15	313.15	323.15
Langmuir			
K_L_ (L·mg^−1^ × 10^−4^)	−7.039	−0.162	−3.922
q_m_ (mg/g)	−13.514	−37.037	−62.893
R^2^	0.992	0.892	0.770
Freundlich			
K_2_ ([mg^1 − (1/n)^/(g·L^−1/n^])	286.00	135.64	90.56
1/n	0.363	0.554	0.649
R^2^	0.995	0.981	0.967
Temkin			
A	6.442	6.195	6.075
B	0.015	0.020	0.021
R^2^	0.980	0.995	0.982

**Table 3:** pH response of IS-pH-SCF under pH from 4 to 8.

pH	L	a	b	∆E	Photos
4	42.228 ± 1.569	48.675 ± 2.211	72.510 ± 2.716	104.503 ± 2.026	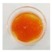
5	28.148 ± 0.614	33.510 ± 0.514	47.930 ± 1.067	92.277 ± 0.264	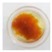
6	0.390 ± 0.064	2.230 ± 0.486	0.398 ± 0.097	85.055 ± 0.052	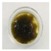
7	0.142 ± 0.004	0.470 ± 0.041	−0.128 ± 0.074	74.276 ± 0.004	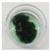
8	0.110 ± 0.001	0.240 ± 0.012	−0.152 ± 0.041	68.307 ± 0.001	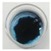
